# Neuroimaging Findings in First Unprovoked Seizures: A Multicentric Study in Tehran

**Published:** 2013

**Authors:** Mohsen MOLLA MOHAMMADI, Seyed Hassan TONEKABONI, Alireza KHATAMI, Eznollah AZARGASHB, Azita TAVASOLI, Mohsen JAVADZADEH, Gholamreza ZAMANI

**Affiliations:** 1 Pediatric Neurology Research Center, Qom University of Medical Sciences, Qom, Iran; 2 Pediatric Neurology Research Center, Shahid Beheshti University of Medical Sciences, Tehran, Iran; 3 Pediatric Neurology Department, Mofid Children Hospital, Faculty of Medicine, Shahid Beheshti University of Medical Sciences, Tehran, Iran; 4 Social Medicine Department, Faculty of Medicine, Shahid Beheshti University of Medical Sciences, Tehran, Iran; 5 Pediatric Neurology Department, Aliasghar Hospital, Iran University of Medical Sciences, Tehran, Iran; 6 Department of Pediatric Neurology, Imam Hossein Hospital, Shahid Beheshti University of Medical Sciences, Tehran, Iran; 7 Department of Pediatric Neurology, Children’s Medical Center, Tehran University of Medical Sciences, Tehran, Iran

**Keywords:** Seizure, First Unprovoked Seizure, Brain Imaging

## Abstract

**Objective:**

Seizure is an emergency in pediatrics. It really matters to the parents of the involved child to have information about the causes, management and prognosis. First unprovoked seizures (FUS) are seizures that occur in patients without fever, trauma or infection. Due to the rapid improvement in diagnostic techniques in the last few decades, the etiology will be revealed and this term will no longer exist. This Study was designed to evaluate brain imaging findings in FUS patients.

**Materials & Methods:**

Ninety-six children with FUS, who were admitted in three major children’s hospitals in Tehran, underwent brain imaging and were enrolled into the study. The decision about the type of imaging (CT or MRI) was based on the patient’s medical and financial conditions. An expert radiologist in the field of pediatric neuroimaging interpreted the images.

**Results:**

Altogether, 27.1% had abnormal findings of which 29.2% were in the brain MRI group and 14.3% were in the brain CT scan group.

Abnormal results were gliosis (10.4%), hemorrhage (4.2%), dysgenesis (2.1%), dysmyelination (7.3%), encephalomalacy (1%), atrophy (5.2%) and infarction (2.1%). In some patients, the lesions were in 2 or 3 sites and some had more than one type of lesion.

There was no association between the duration, age and type of seizure and imaging abnormlities. However, we found an association between the location of the lesion and the type of seizure.

**Conclusion:**

We recommend brain imaging in all patients with FUS and apart from some exceptions, brain MRI is superior to CT.

## Introduction

Seizure is a common neurologic problem that occurs in 3-5% of children (1).

First unprovoked seizure (FUS) is a type of seizure with no obvious precipitating cause (2), but improvement of the diagnostic techniques increases the chance of finding the unknown causes.

Imaging studies are one of the modalities that are very important in the management of this abnormality revealing abnormal findings more common than before. Abnormal imaging findings which are mentioned as 10% (3,4), 21% (5) and 31% by Kalnin etal (6) are seen in many studies. 

In a study conducted by Mark King (7) in 1998 on 300 cases with FUS, they found an epileptogen lesion in 38 cases; consequently suggesting imaging in FUS cases. Pohlmam-Eden et al (8) also emphasized the need for brain MRI in all adults with the first epilepsy and all children with seizure except those with an idiopathic syndrome or the genetic focal or generalized type. They also confirmed that brain MRI has advantage over brain CT scan. However, other investigators considered special situations such as abnormal neurological signs, history of malignancy, coagulative disorders and infants less than 3 months of age for imaging (9). 

Researchers (2, 10, 11) recommended brain imaging in FUS; however, not in the emergency room, but imaging in the following days of seizures is necessary. Daniel (12) in 2011 pointed to the fact that brain MRI increased hospitalization and the patients’ costs. On the other hand, imaging findings may not change the patient’s treatment protocol; so, he recommended brain MRI on an outpatient basis. William Gaillard (13) also highlighted brain imaging in cases of FUS for localization, characterization and emergent intervention, if necessary. 

So we decided to carry out a survey as a systematic evaluation to find the abnormal imaging findings in Iranian cases.

## Materials & Methods

We evaluated 96 cases admitted to Shahid Beheshti and Tehran university-affiliated hospitals with first unprovoked seizure (FUS). All cases were sent for imaging, All the imagings were performed by 1.5 Tesla MRI or 16-slice CT scan Siemens. 

Sedation was given if needed to reduce motion artifacts (14). All our cases were hospitalized due to first seizure episodes. Eleven cases who refused hospitalization were excluded from the study.

The entire imagings were evaluated by an experienced pediatric radiologist and reassessed in cases of doubt in a neuroradiological meeting with child neurologists. 

All of the clinical and paraclinical data were included in a figure for further evaluation. Imaging findings such as mass, encephalomalacia, porencephaly, gliotic and demyelinating lesions, the location of the lesion with demographic data such asgender, age, duration of the seizure, type of seizure, past medical history and labor history were included.

The main including criteria were: 1-first seizure. 2- no fever. 3- no recognized drug usage. 4-no evidence of CNS infection. 5- no background or evidence of previous seizure. 6- no neurological sign. 7- age older than 1 month.

This study was a cross sectional descriptive-analytic study. The data were analyzed by SPSS version 16 using t-test, Fisher’s test, Kolmogorov-Smirnov, Mann- Whitney and Chi square tests. A p value lower than 0.05 was considered as statistically significant.

## Results

**Chart 1:**
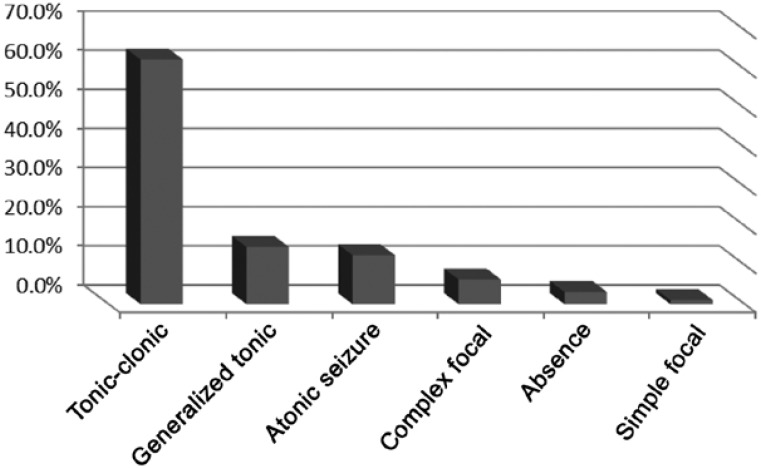
Distribution of seizure type in children with FUS

Ninety-six cases [of which 53 (55.2%) were male] were included in the study. The patients’ mean age was 68/7±48/2 (range, 2-180) months. According to the type of seizure,s ix groups; namely, generalized tonicclonic 60/96 (62.5%), generalized tonic 14/96 (14.6%), atonic seizure 12/96 (12.5%), absence 3/96 (3.1%), focal complex 6/96 (6.3%) and simple focal 1/96 (1%) were categorized (Chart 1). 

Sixty-eight of 96 cases (70.8%) had no family history of seizure. The mean duration of the seizures was 4.6 minutes (range, 0.4-20 minutes) and none of the cases had a prenatal or perinatal history of hemorrhage or asphyxia.

**Chart 2:**
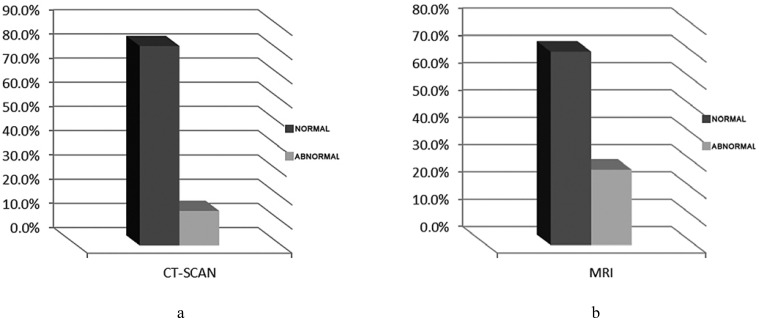
a,b. Distribution of imaging findings in children with FUS

**Chart 3:**
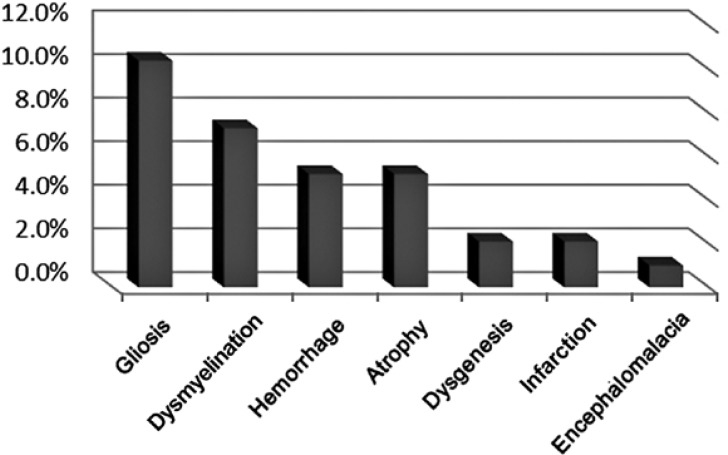
Distribution of lesion types in brain imaging

**Fig 1:**
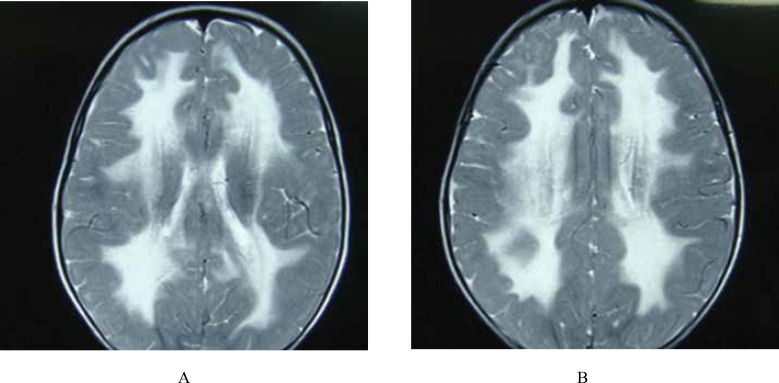
An 18-Month-Old Boy With FUS: Axial T2W (A & B) images reveal bilateral hypersignal white matter changes due to leukodystrophy

**Fig 2:**
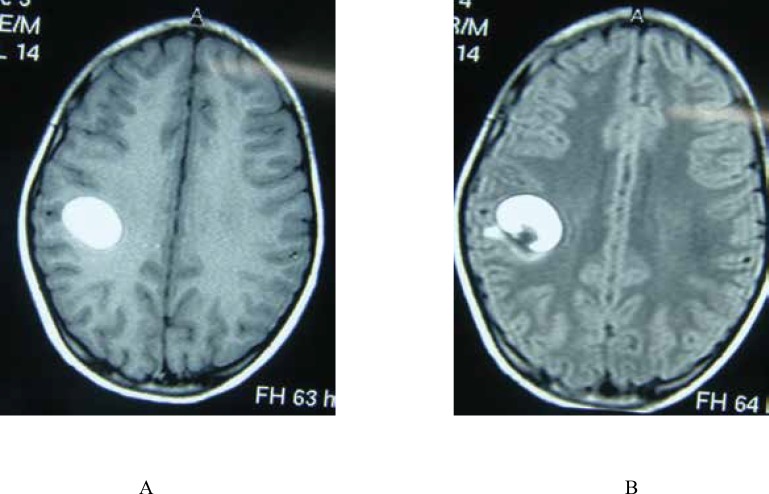
An 11-Year-Old Boy With FUS : T1W (A) and FLAIR (B) images reveal high signal intensity lesion in the left parietal lobe with hyposignal internal component due to hematoma

Eighty-two cases (85.4%) underwent MRI and 14 cases (14.6%) underwent CT scan. Twenty-six of the patients (27.1%) had an abnormal imaging. 24 cases with MRI (29.27%) had abnormality and 2 of 14 cases with CT scan (14.3%) had abnormal findings (Chart 2 a,b).The most common imaging findings were gliosis in 10 (10.4%), dysmyelination 6 (7%) (Figure 1), hemorrhage in 5 (5.2%) (Figure 2) and brain dysgenesis in two (2%) (Chart 3).

**Fig 3:**
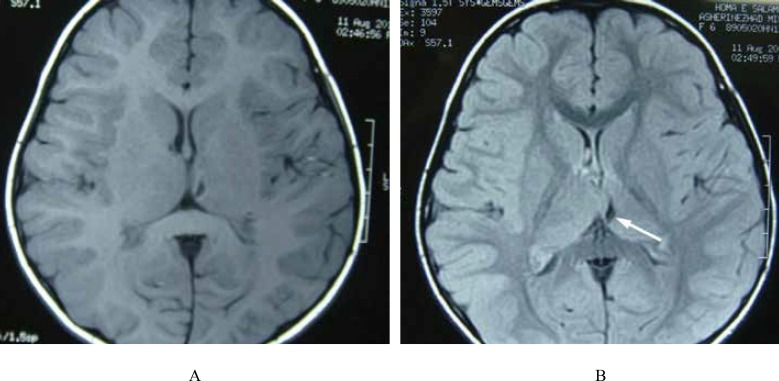
A 7-Year-Old Girl With FUS: Axial T1W (A&B) images through thalami reveal porencephalic lesion in the left thalamus due to an old infarct

**Fig 4:**
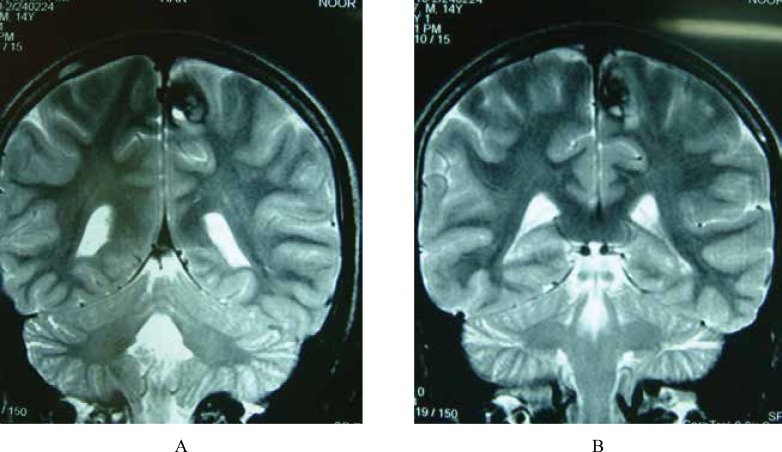
A 13 year-Old-Boy With FUS; Coronal T2W (A & B) images through occipital horns reveal signal void left parasagittal lesion due to A-V malformation

The location of brain involvement was a white matter lesion in 10 cases (10.4%), corpus callosum in three (3.1%), thalamus in one (1%) (Figure 3) and basal ganglia in two (2.1%). Other focal lesions were seen in the frontal, parietal (Figure 4), temporal lobes and the cerebellum in one (1%), four (4.2%), nine (9.4%) and two (2/1%) cases, respectively. 

**Chart 4:**
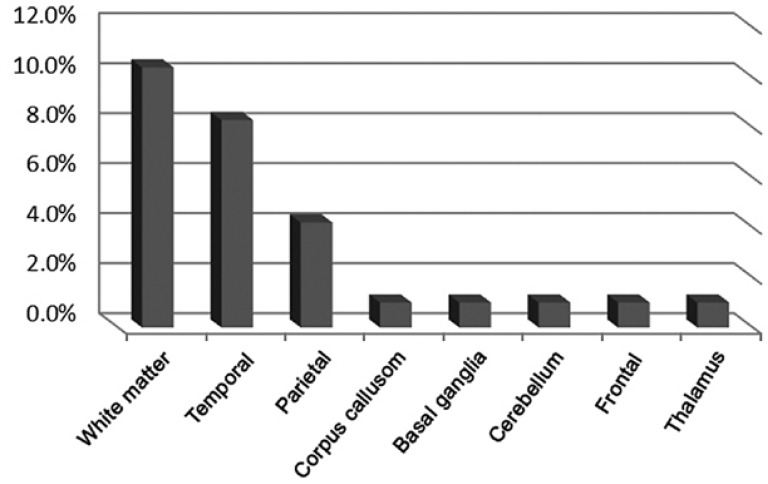
Distribution of lesion location in brain imaging of children with FUS

We had no lesion in the ventricular system, midbrain, pons or occipital region (Chart 4). 

Although seven (7.3%) cases had more than one location and more than one kind of lesion. We had electroencephalographic abnormality in 29 (30.2%) cases. 

Evaluation for any correlation between the findings was carried out. 

MRI revealed more lesions than CT scan; however, no significant difference is noticed (p=0.286). There was also no difference between the type of seizure and imaging modality for the detection of abnormality; though generalized tonic-clonic (GTC) was the most common seizure pattern detected (p=0.611).

Otherwise more common findings are descriptive in this study without significant correlation.

## Discussion

In retrospective studies by Khodapanahandeh (3) in Iran and Alawaneh (4) in Saudi Arabia, a 10% abnormality in FUS cases was detected, although in their study no distinction was made between brain CT scan and MRI in their patients. Our study with 14% abnormal CT finding revealed more abnormal imaging findings than this study. In a five-year study from 1999 to 2004, conducted by Khodapanahandeh, five brain hemorrhages followed by brain tumor, tuberous sclerosis, SLE, ischemia, arachnoid cyst and ADEM were detected, all of which consisted of one case. Shlomo (5) in another study found 21% abnormality. He also found that six cases with normal brain CT scan had migration anomaly in further MRIs and he too recommended brain imaging for FUS patients.

Beverly (10) found EEG focal abnormality and suggested focal abnormal EEG may be used as a marker for brain imaging; nonetheless, our work revealed no significant association which was also confirmed by Jason(15).

Andrew Kalnin (6) found abnormality in 31% of the patients’ imagings and similar to Daniel(12) and Beverly (10), he recommended imaging for all cases of FUS. 

Follow up and data reveal 50% of the patients with FUS showed only one time seizure and had no experience of recurrence seizure unrelated to underlying brain anomaly. But findings abnormal brain is helpful for better management of these patients. 

In Khodapanahandeh’s study (3), the patients’ ages were 1 month to 15 years (54.4% female, 45.6% male). The mean age was 53 months.

Shinnar (5) in a 10-year prospective study on 411 cases with FUS doing imaging in 218 cases (53%). 159 CT scan and 59 brain MRI. Only 45 cases (21%) of 218 cases had abnormal findings which smaller than our work (27/1%).Indeed four of these cases needed immediate surgical intervention due to brain tumor however, non of our cases needed surgical intervention.

According to Kings (7), 12.7% cases of FUS revealed imaging abnormality in the brain MRI which was also smaller that what are found. 

In 2010, Bano (11) revealed that finding structural or metabolic abnormalities which need special treatment are the main purpose of imaging in FUS. He recommended CT scan for emergency situations, although the sensitivity is as low as 30%.

In a study by Kalnin (6) in 2008 on 281 (age, 6 to 14 years old)patients, he showed a 31% abnormality with at least one brain lesion, of which 12% had two or more lesions. 

The most common lesions were ventriculomegaly (51%), gliosis (23%), dysplasia or heterotopias in 12%, brain atrophy in 12%, white matter disease in 9% and encephalomalacia in 6% which was different from what we found.

Jasone (15) in 181 cases revealed that 32/6% of the cases had abnormal MRIs. They also noted that when they found normal EEGs in 50 cases with FUS, there was 42% abnormal MRI findings(21 cases). This finding is supported by our study.

**Suggestions**

1) Imaging as a main diagnostic tool must be performed for every case with FUS.

2) MRI with the accuracy of depicting white matter abnormalities and myelinating disorders has advantage over CT scan, especially in children older than 6 months of age. On the other hand, white matter abnormality is the leading cause of abnormality in our study.
